# Cortical somatosensory processing after botulinum toxin therapy in post-stroke spasticity

**DOI:** 10.1097/MD.0000000000026356

**Published:** 2021-06-25

**Authors:** Tomáš Veverka, Petr Hluštík, Pavel Otruba, Pavel Hok, Robert Opavský, Jana Zapletalová, Petr Kaňovský

**Affiliations:** a Department of Neurology, Palacký University Olomouc and University Hospital Olomouc; b Department of Biophysics, Biometry and Statistics, Palacký University Olomouc, Czechia.

**Keywords:** botulinum toxin, somatosensory cortex, somatosensory evoked potentials, spasticity, stroke

## Abstract

In dystonic and spastic movement disorders, abnormalities of motor control and somatosensory processing as well as cortical modulations associated with clinical improvement after botulinum toxin A (BoNT-A) treatment have been reported, but electrophysiological evidence remains controversial. In the present observational study, we aimed to uncover central correlates of post-stroke spasticity (PSS) and BoNT-A-related changes in the sensorimotor cortex by investigating the cortical components of somatosensory evoked potentials (SEPs). Thirty-one chronic stroke patients with PSS of the upper limb were treated with BoNT-A application into the affected muscles and physiotherapy. Clinical and electrophysiological evaluations were performed just before BoNT-A application (W0), then 4 weeks (W4) and 11 weeks (W11) later. PSS was evaluated with the modified Ashworth scale (MAS). Median nerve SEPs were examined in both upper limbs with subsequent statistical analysis of the peak-to-peak amplitudes of precentral P22/N30 and postcentral N20/P23 components. At baseline (W0), postcentral SEPs were significantly lower over the affected cortex. At follow up, cortical SEPs did not show any significant changes attributable to BoNT-A and/or physiotherapy, despite clear clinical improvement. Our results imply that conventional SEPs are of limited value in evaluating cortical changes after BoNT-A treatment and further studies are needed to elucidate its central actions.

## 1 Introduction

Post-stroke spasticity (PSS) is one of the main motor consequences of stroke.^[1]^ Severe PSS lowers the patient's quality of life and frequently causes significant limitations of gross and fine motor control, gait/falling, and activities of daily living (ADL).^[2]^ Recommended treatment regimens to alleviate PSS combine physiotherapy procedures and botulinum toxin A (BoNT-A) applications.^[3–5]^ BoNT-A has been proven to be safe and effective in relieving upper limb PSS and improving motor functions.^[6,7]^ BoNT-A produces its therapeutic effects primarily by inhibiting acetylcholine release from the pre-synaptic terminals of the alpha motoneurons on muscle spindles.^[8–10]^ Besides this peripheral site of action, central effects have been reported as well.^[11,12]^ Effects of BoNT-A injected in the periphery likely spread through supraspinal mechanisms and may even cause reorganization of the cerebral cortex.^[13]^ Various neurophysiological techniques have been applied to investigate BoNT-A-related modulation of the sensorimotor cortex.^[14]^ Somatosensory evoked potentials (SEPs) have been established as an appropriate method for assessing the integrity of sensory-motor pathways and studying the effect of the afferent peripheral inputs on the sensorimotor cortex.^[15]^ Several studies have shown that cortical components of SEPs are altered in stroke patients and they may even have a prognostic value in predicting recovery of the upper limb function.^[16,17]^ More interestingly, there is evidence that BoNT-A application not only leads to improvement of spasticity but is also associated with the normalization of impaired cortical SEPs.^[18–20]^ However, despite these results, it is still under debate whether cortical SEPs are sensitive enough to detect the central (remote) effects of BoNT-A treatment.^[14]^

The purpose of the present study was to uncover central correlates of PSS and BoNT-A-related changes in the sensorimotor cortex, investigating the cortical components of SEPs. We hypothesized that pathological SEPs would be recorded over the lesioned hemisphere and that they would partially normalize after BoNT-A application and physiotherapy. A longitudinal study protocol with three time-points was designed to separate the effects of BoNT-A application from the effects of physiotherapy.

## 2 Methods

### 2.1 Patients

The protocol of the observational study was approved by the Ethics committee of University Hospital Olomouc and was conducted in accordance with the tenets of the Declaration of Helsinki. All subjects provided their written consent before participating in this study. Thirty-one right-handed chronic stroke patients (20 males and 11 females) with clinically relevant PSS of the upper limb and at least three months since stroke were recruited from the Comprehensive Stroke Center at the Department of Neurology, University Hospital, Olomouc, Czechia. The mean age at study entry was 59 ± 14.9 (SD) years. The patients’ demographic characteristics are listed in Table 1. Enrolled subjects were required to be in the chronic stage of first-ever ischemic stroke; the time from stroke onset to the study entry ranged from 3 to 139 months, the median was 10 months. The ischemic lesions, confirmed by magnetic resonance imaging or computed tomography, were subcortical or cortico-subcortical within the middle cerebral artery territory. Hand spasticity was clinically relevant and exceeded 1 on the modified Ashworth scale (MAS).^[21]^ Exclusion criteria were: history of BoNT-A application or drugs affecting muscle hypertonus intake; contraindications for BoNT-A application; and SEPs exclusion criteria (i.e., implanted electronic devices). All subjects underwent clinical and electrophysiological evaluation just before BoNT-A application (week 0, W0), 4 weeks later (W4), and 11 weeks later (W11). A longitudinal within-subject study design was used, in which each patient served as their own internal control.

**Table 1:** Demographic and clinical characteristics.

Patient	Age	Sex	Stroke to W0 (months)	Affected hand	mRS	BI	mMRC (WF/WE, FF/FE)	Global MAS W0	Global MAS W4	Global MAS W11
1	64	F	6	R	3	70	0/0,0/0	2	1.25	1.75
2	68	M	7	L	2	90	4/4,4/4	2	1.25	2
3	51	F	23	R	3	65	0/0,0/0	3	2	2
4	75	F	10	R	3	85	1/1,2/1	2	1	2
5	22	M	3	R	3	85	0/0,0/0	3	1	2.5
6	25	F	11	L	3	80	1/0,0/0	2.5	1.5	2.5
7	60	M	9	L	2	90	4+/4,4+/4	3	2	2.5
8	77	M	18	R	2	70	4/3,4/3	3	1.75	3
9	74	F	3	L	4	40	0/0,0/0	3	2	3
10	54	M	15	R	2	85	4/3,4/3	1.5	2	2
11	62	M	139	L	3	90	0/0,0/0	2.5	1.5	2.25
12	69	F	9	R	3	85	0/0,0/0	2.5	1.5	2
13	69	M	9	R	3	90	0/0,0/0	2.5	2	2.5
14	66	M	4	L	3	80	3/2,3/2	3	1.75	2.5
15	71	M	76	R	3	95	3/2,3/2	2.5	1.75	2.5
16	66	M	14	R	3	85	2/1,2/1	2	2	2
17	51	F	19	L	4	75	0/0,0/0	3	1.25	2
18	72	M	23	R	3	100	3/3,2/3	1.5	1	1
19	33	M	32	L	3	70	2/1,3/1	2	1	2
20	44	M	3	L	2	100	4/3,4/3	1.5	0.5	0.5
21	31	M	7	L	3	90	1+/0,1+/1+	3	1.5	2.5
22	67	F	5	R	4	60	0/0,0/0	3	2	3
23	63	M	38	L	4	65	1/0,1/0	3	3	3
24	49	M	43	L	2	100	4/3,4/3	2	0.5	0.5
25	60	M	21	R	3	75	0/0,0/0	3	2	2.5
26	72	M	15	R	4	60	4/3,4/3	2	0.5	2
27	71	F	18	L	2	90	4/3,4/3	2	0.5	2
28	55	F	10	R	3	85	2/0,2/0	2.5	2	1.75
29	55	M	9	L	3	90	3/2,3/0	2	1.25	2
30	69	M	4	R	2	80	3/2,3/2	2	1.5	2
31	70	F	8	R	3	95	0/0,0/0	2	1.75	2.5

BI = Barthel Index, FE = finger extensors, FF = finger flexors, L = left, MAS = modified Ashworth scale, mMRC = modified Medical Research Council scale, mRS = modified Rankin Scale, R = right, WE = wrist extensors, WF = wrist flexors.

### 2.2 Clinical evaluation

PSS was evaluated using the MAS at each visit. The MAS was scored separately for fingers and wrists and the values were averaged together (global MAS score). For statistical analysis, a grade of 1+ on the MAS was recorded as 1.5. Further clinical investigations included the following standardized scales performed at W0: the modified Medical Research Council scale^[22]^ to test upper extremity strength and the Barthel Index^[23]^ and the modified Rankin Scale^[24]^ to assess disability. The clinical characteristics of the subjects are listed in Table 1.

### 2.3 Treatment

All subjects were treated with BoNT-A injections into the spastic muscles of the affected arm at W0 followed by a dedicated physiotherapy protocol.^[25,26]^ The injections were performed using EMG guidance (Medtronic Keypoint, Alpine Biomed ApS, Denmark), preferably using electrical stimulation to localize the target. The following muscles were always injected: flexor carpi ulnaris, flexor carpi radialis, flexor digitorum superficialis, and flexor digitorum profundus. Each muscle was consistently injected with BoNT-A in a fixed dose of 50 UA (BOTOX; Allergan, Inc., Irvine, CA, USA) in accordance with current recommendations.^[5,27]^ Physiotherapy started several days after the BoNT-A injection (W0). Initial inpatient physiotherapy lasting for 2–4 weeks was followed by outpatient physiotherapy until the third clinical evaluation (total of 11 weeks). Patients underwent daily physiotherapy sessions for 1 h on weekdays, that is, five times per week. Individual kinesiotherapy included posture-locomotion training towards restitution of bipedal posture and gait, motor recovery of the hips and trunk using elements of the Bobath concept, proprioceptive neuromuscular facilitation, respiratory physiotherapy, reflex and myofascial techniques, anti-spastic positioning, occupational therapy, and training of independence in ADL. Proper adherence to the physiotherapy protocol was checked at each examination throughout the study period.

### 2.4 Somatosensory evoked potentials (SEPs)

Median nerve SEPs were examined in both upper extremities using the Keypoint device (Medtronic, Dublin, Ireland). We used a previously published protocol for recording and stimulation.^[15,28]^ Both median nerves were consecutively stimulated at the wrist. Square-wave pulses lasting 0.1 ms were used at an intensity that was 1.5 times higher than the motor threshold that evoked thumb twitching. A 5-Hz stimulation frequency was used in all examinations. The cortical components of SEPs were recorded over the contralateral somatosensory cortex by using surface silver-silver chloride electrodes in C3+, C4+, C3’, and C4’ electrode positions according to the International 10–20 system. C3+ and C4+ were placed 2 cm posterior to C3 and C4; C3’ and C4’ were placed 2 cm anterior to C3 and C4. Mutually connected earlobes were used as a reference. Skin resistance was maintained at 4 kΩ and was checked repeatedly during each recording session. The responses were filtered using a bandpass filter of 10–2000 Hz, and the time base was 50 ms. Analysis time was 5 ms/division. Two runs of 500 artefact-free sweeps were averaged in each recording session. The peaks were labelled according to the nomenclature published by Donchin et al^[29]^ Peak-to-peak amplitudes of postcentral N20/P23 (at C3+ and C4+ electrodes) and precentral P22/N30 (at C3’ and C4’ electrodes) components were then measured in the superimposed runs and subsequently statistically analysed. To prevent the baseline shift bias, the absolute values of the N20, P23, P22, and N30 were not used. Side-to-side ratios of N20/P23 and P22/N30 amplitudes (affected/unaffected side) were then calculated. Additionally, absolute latencies for N20 (measured from the beginning of the stimulus to the maximal level of negative deflection) were recorded.

### 2.5 Statistics

All statistical analyses were carried out using IBM SPSS Statistics for Windows, version 22.0 (IBM Corp., Armonk, NY, USA). The Wilcoxon signed-rank test with Bonferroni correction was used to compare global MAS scores from W0, W4, and W11. Differences in SEPs of affected and unaffected side at W0 were analysed using the Wilcoxon signed-rank test. Changes in SEPs at W4 (BoNT-A-effect) and W11 (effect of physiotherapy), as compared to W0, were tested using the Friedman test. Normal distribution was verified by the Shapiro–Wilk test. A *P*-value less than .05 was considered statistically significant (*P* < .05).

## 3 Results

### 3.1 Clinical

**Figure 1:**
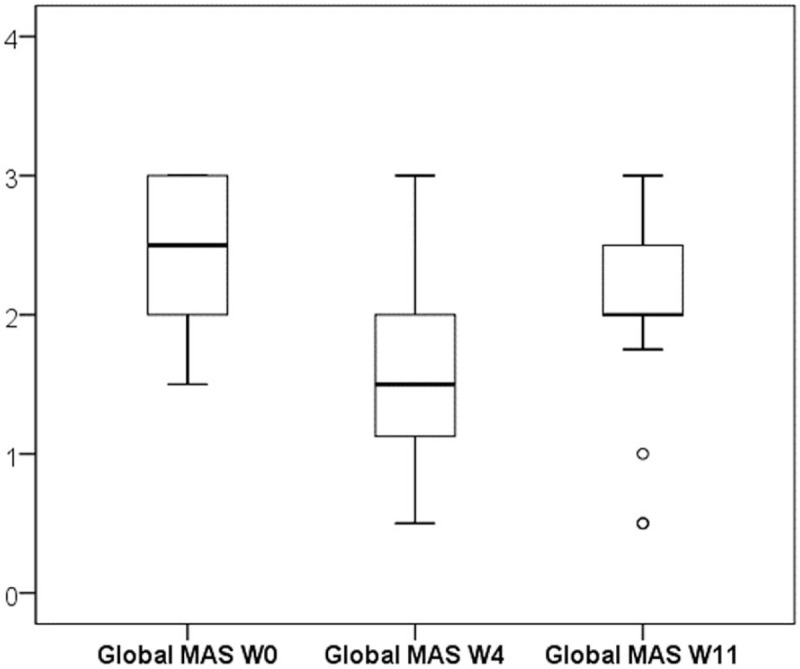
Effect of BoNT-A treatment on global MAS scores. The edges of the box represent the 25th and 75th percentiles, the horizontal thick line inside the box represents the median, and the whiskers represent the maximum and minimum values.

Treatment with BoNT-A and subsequent physiotherapy significantly reduced PSS of the upper limb. Global MAS showed statistically significant decreases at W4 (*P* < .0001) with subsequent increases at W11 (*P* = .013), although the reduction against W0 remained significant (*P* = .0001). The median global MAS scores were 2.50 at W0 (interquartile range (IQR) = 2.0–3.0), 1.50 at W4 (IQR = 1.13–2.00), and 2.00 at W11 (IQR = 2.0–2.5). Individual MAS scores for each subject are listed in Table 1 and statistical data are illustratively presented in a box plot in Figure 1.

### 3.2 SSEP

At the baseline (W0), the peak-to-peak amplitude of postcentral N20/P23 during stimulation of the impaired limb was significantly lower in comparison to the unaffected side (*P* = .003). The peak-to-peak amplitude of precentral P22/N30 and the absolute latency for N20 did not significantly differ between the affected and unaffected sides (Table 2).

**Table 2:** Somatosensory evoked potential values at the baseline (W0).

	Mean	SD	Median	Min	Max	Wilcoxon test *P*
N20 latency, unaffected limb (ms)	20.85	1.68	20.90	17.40	24.10	.729
N20 latency, affected limb (ms)	20.93	1.53	21.00	18.40	25.70	
P22/N30, unaffected limb (μV)	1.74	1.40	1.43	0.04	5.78	.308
P22/N30, affected limb (μV)	1.49	1.17	1.34	0.15	5.24	
N20/P23, unaffected limb (μV)	**2.95**	2.33	**2.44**	0.47	9.67	**.003**^∗^
N20/P23, affected limb (μV)	**1.73**	1.94	**1.10**	0.17	8.75	

SD = standard deviation.

∗ Statistically significant difference (*P* < .05).

After the treatment, none of the assessed cortical SEP values (postcentral N20/P23, precentral P22/N30, side-to-side ratios of N20/P23 and P22/N30, and absolute latencies for N20) yielded statistically significant treatment-related changes despite clear clinical improvement (Table 3).

**Table 3:** Changes in somatosensory evoked potential values at W4 (BoNT-A-effect) and W11 (effect of physiotherapy), against W0.

	Mean	SD	Median	Min	Max	Friedman test *P*
Affected limb						
N20 latency W0 (ms)	20.93	1.53	21.00	18.40	25.70	.215
N20 latency W4 (ms)	21.27	2.09	21.00	17.60	26.20	
N20 latency W11 (ms)	20.96	1.90	21.00	18.20	25.60	
P22/N30 W0 (μV)	1.49	1.17	1.34	0.15	5.24	.241
P22/N30 W4 (μV)	1.06	0.62	0.95	0.19	3.03	
P22/N30 W11 (μV)	1.36	1.16	0.97	0.10	6.05	
N20/P23 W0 (μV)	1.73	1.94	1.10	0.17	8.75	.857
N20/P23 W4 (μV)	1.51	1.46	0.86	0.23	7.49	
N20/P23 W11 (μV)	1.78	1.85	0.79	0.11	6.95	
Unaffected limb						
N20 latency W0 (ms)	20.85	1.68	20.90	17.40	24.10	.512
N20 latency W4 (ms)	21.00	1.76	20.30	17.10	24.10	
N20 latency W11 (ms)	20.66	1.49	20.40	17.90	24.10	
P22/N30 W0 (μV)	1.74	1.40	1.43	0.04	5.78	.879
P22/N30 W4 (μV)	1.71	1.27	1.11	0.13	5.23	
P22/N30 W11 (μV)	1.65	1.25	1.30	0.35	6.02	
N20/P23 W0 (μV)	2.95	2.33	2.44	0.47	9.67	.062
N20/P23 W4 (μV)	3.75	2.89	2.78	0.74	14.23	
N20/P23 W11 (μV)	3.12	2.16	2.63	0.51	9.96	

SD = standard deviation. Statistically significant difference (*P* < .05).

## 4 Discussion

In the present study we aimed to uncover central correlates of PSS and BoNT-A-related changes in the sensorimotor cortex by investigating the cortical components of SEPs. Our results revealed the expected decrease of SEP amplitude over the postcentral sites in the lesioned hemisphere; however, we did not find the hypothesized change of SEP parameters associated with BoNT-A therapy.

As expected, BoNT-A and physiotherapy effectively improved PSS. There was a significant decrease in the global MAS score at W4 and W11, with the maximal effect at W4, when the pharmacological peripheral effect of BoNT-A is assumed to be highest. This finding is in line with other studies evaluating BoNT-A efficacy in PSS.^[30]^ At W11, the global MAS score significantly increased but remained lower than at the baseline. Similarly, as in our recent functional magnetic resonance imaging (fMRI) study in an almost identical cohort of patients, some improvement of PSS persisted at the follow-up visits, even though the local BoNT-A effect should have waned.^[31]^ This observation could be explained by ongoing physiotherapy, but the theory of persistent central reorganization after BoNT-A should also be considered.^[12]^ A prolonged clinical effect of BoNT-A application, exceeding the average duration of the neuromuscular blockade, has been observed in clinical routine in a number of patients with spastic or dystonic disorders.^[32,33]^

Most of the neurophysiological evidence for distant effects of BoNT-A comes from studies with dystonic patients. Unlike PSS, with its well-defined lesion of descending tracts, dystonic disorders have different pathophysiology with no morphological impairment of the central nervous system (CNS). The putative mechanism by which BoNT-A injected in the periphery may induce dynamic changes at several hierarchical levels of the sensorimotor system, presumably including the cerebral cortex, was first postulated in cervical dystonia.^[15]^ Despite substantial differences, the same plasticity mechanism is also presumed in spasticity. Besides its effect on extrafusal muscle spindles, BoNT-A alters pathological proprioceptive flow from intrafusal fibres through the Ia afferents to the CNS and indirectly modulates the sensorimotor cortex.^[10,34]^ Kaňovský et al reported a higher amplitude of precentral P22/N30 and the normalization of this SEP component after BoNT-A treatment in cervical dystonia.^[15]^ The authors concluded that the increased P22/N30 amplitude likely reflects abnormally enhanced cortical excitability and that BoNT-related change in amplitude might be a consequence of the normalization of this excitability. It should be noted that no significant abnormality in the postcentral component (N20/P23) was found in that study. Further studies reported impairment of both cortical excitability and intracortical inhibition in focal dystonia, as well as normalization following BoNT-A injection.^[35,36]^ However, subsequent studies did not confirm normalized cortical excitability following BoNT-A therapy.^[37,38]^ Similarly, no changes in SEPs before and after BoNT-A were reported by Contarino et al in patients with writer's cramp.^[39]^ Moreover, SEP amplitudes and latencies in patients with writer's cramp did not differ from those of healthy controls.

In focal spasticity, several earlier studies focused on cortical SEPs and electrophysiology changes with BoNT-A treatment. Park et al and Frascarelli et al reported flat or abnormal SEPs in children with spastic cerebral palsy and improvement in SEPs after BoNT-A application.^[18,19]^ They concluded that spasticity itself affects cortical SEPs and improvement in SEP parameters associated with reduced spasticity is likely related to a central reorganization. Another study by Basaran et al investigating patients with PSS showed longer N20 latency and lower N20-P25 in the affected limb compared to the unaffected side. However, even though BoNT-A led to improvement in SEPs, the difference did not reach statistical significance, likely due to small sample size.^[20]^

In our present study, prior to treatment the peak-to-peak amplitude of N20/P23 during the stimulation of the impaired limb was significantly lower than on the unaffected side, which agrees with the aforementioned studies. Abnormal or absent cortical SEP responses evoked from the affected limb are common in most stroke patients;^[16]^ therefore, SEP abnormalities after stroke cannot be attributed to spasticity alone.

Regarding the main aim of our study, cortical SEPs did not show any significant changes attributable to BoNT-A and/or physiotherapy. After the treatment, although there was clear clinical improvement, none of the assessed cortical SEP values yielded statistically significant treatment-related changes. These findings are in line with our previous study, which had a similar design and a smaller number of patients.^[28]^ The only study that reported improvement in SEPs after BoNT-A application in PSS was preliminary and conducted in a small cohort of spastic patients, which limits its overall impact.^[20]^

Our results, in a large group of patients with PSS, may be explained by several considerations. First, the central effects of BoNT-A may not involve primary somatosensory cortical responses as recorded by our SEP protocol. In our recent neuroimaging study using fMRI, we demonstrated that BoNT-A treatment of PSS is associated with transient changes in the ipsilesional posterior parietal cortex (PPC).^[31]^ Decreased activation in the PPC after treatment probably reflects a change in the internal model of the subject's hand resulting from decreased proprioceptive flow from the spastic limb. However, neurophysiological exploration of the parietal cortex behind the primary somatosensory cortex is beyond the scope of the early evoked cortical components used in the present study.

Second, there is still some debate on whether cortical SEPs evoked by electrical stimulation are sensitive and specific enough to assess the central (remote) effects of BoNT-A treatment.^[14]^ Natural proprioceptive afferent stimulation, which is the presumed signal mediating the central effects of BoNT-A,^[40]^ may be significantly different from artificial electrical stimulation of a mixed peripheral nerve.

Third, hemispheric asymmetry and high variability in SEP amplitudes following median nerve stimulation, reported even in healthy subjects,^[41,42]^ are likely to contribute to the lack of significant BoNT-A-related changes in PSS patients.

In dystonic and spastic movement disorders commonly treated with BoNT-A, abnormalities of motor control and somatosensory processing have been reported, as well as cortical modulations associated with clinical improvement after BoNT-A treatment,^[31,33,43,44]^ but electrophysiological evidence (SEPs) remains controversial.

We acknowledge that the patient sample size is modest and larger patient group may be needed to achieve robust SEP results. A bigger patient sample might also better reflect the male-to-female ratio expected in the general stroke patient population. The patient selection involved ischemic strokes with dominant spastic hemiparesis as defined in our inclusion criteria, however, the ischemic lesions did differ in location and size.

Future studies may include coverage of a broader parietal area and investigation of the parietal P100, which represents activation of the secondary somatosensory cortex and the posterior parietal cortex.^[45]^ Alternative modes of somatosensory stimulation may be more sensitive to capturing the afferentation presumably altered by BoNT-A.^[40]^

## 5 Conclusions

In PSS, median nerve SEPs manifest the expected decrease of SEP amplitude over the lesioned hemisphere. In contrast to recent fMRI findings, there were no significant changes associated with BoNT-A treatment and physiotherapy applied for more than three months. Future studies with altered methodology may be needed to detect electrophysiological correlates of spasticity therapy.

## Author contributions

**Conceptualization:** Petr Hluštík, Petr Kaňovský.

**Data curation:** Tomáš Veverka.

**Formal analysis:** Jana Zapletalová.

**Investigation:** Pavel Otruba.

**Methodology:** Robert Opavský.

**Supervision:** Petr Kaňovský.

**Writing – original draft:** Tomáš Veverka.

**Writing – review & editing:** Petr Hluštík, Pavel Hok.
